# Report on First International Workshop on Robotic Surgery in Thoracic Oncology

**DOI:** 10.3389/fonc.2016.00214

**Published:** 2016-10-24

**Authors:** Giulia Veronesi, Robert Cerfolio, Roberto Cingolani, Jens C. Rueckert, Luc Soler, Alper Toker, Umberto Cariboni, Edoardo Bottoni, Uberto Fumagalli, Franca Melfi, Carlo Milli, Pierluigi Novellis, Emanuele Voulaz, Marco Alloisio

**Affiliations:** ^1^Thoracic Surgery, Humanitas Research Hospital, Rozzano, Milan, Italy; ^2^Thoracic Surgery, University of Alabama at Birmingham, Birmingham, USA; ^3^Istituto Italiano Tecnologia, Genova, Italy; ^4^Universitätsmedizin Berlin – Charité Campus Mitte, Berlin, Germany; ^5^IRCAD, Strasburg, France; ^6^Department of Thoracic Surgery, Istanbul Bilim University, Istanbul, Turkey; ^7^General Surgery, Humanitas Research Hospital, Rozzano, Milan, Italy; ^8^Chirurgia Toracica, Ospedale Cisanello, Pisa, Italy; ^9^Direzione amministrativa, Azienda Ospedaliera Cisanello, Pisa, Italy

**Keywords:** robotic surgery, lung cancer, segmentectomy, virtual reality, cost–benefit analysis

## Abstract

A workshop of experts from France, Germany, Italy, and the United States took place at Humanitas Research Hospital Milan, Italy, on February 10 and 11, 2016, to examine techniques for and applications of robotic surgery to thoracic oncology. The main topics of presentation and discussion were robotic surgery for lung resection; robot-assisted thymectomy; minimally invasive surgery for esophageal cancer; new developments in computer-assisted surgery and medical applications of robots; the challenge of costs; and future clinical research in robotic thoracic surgery. The following article summarizes the main contributions to the workshop. The Workshop consensus was that since video-assisted thoracoscopic surgery (VATS) is becoming the mainstream approach to resectable lung cancer in North America and Europe, robotic surgery for thoracic oncology is likely to be embraced by an increasing numbers of thoracic surgeons, since it has technical advantages over VATS, including intuitive movements, tremor filtration, more degrees of manipulative freedom, motion scaling, and high-definition stereoscopic vision. These advantages may make robotic surgery more accessible than VATS to trainees and experienced surgeons and also lead to expanded indications. However, the high costs of robotic surgery and absence of tactile feedback remain obstacles to widespread dissemination. A prospective multicentric randomized trial (NCT02804893) to compare robotic and VATS approaches to stages I and II lung cancer will start shortly.

## Introduction

The first day of the Workshop consisted mainly presentations and discussions in the broad areas of *future evolution of medical robotics, costs, current situation of robotics in thoracic surgery*, and *research perspectives in robotic thoracic surgery*. The second day was mainly concerned with the presentation of live surgery sessions, with ample time for questions and discussion.

## Robotic Surgery for Lung Resections

Thoracic surgeons should only consider starting to use a robot if they are enthusiastic about the technique: the fact that the hospital has bought robots and was setting up a robot unit is not sufficient reason for getting involved. Thoracic surgeons should be enthusiastic about robotic surgery because of its high-definition visibility, greater independence, and easier more intuitive movements compared to video-assisted thoracoscopic surgery (VATS). In addition, robotic surgery has advantages for the patient. These are similar to those afforded by VATS (1) and include reduced pain, fewer complications, and shorter postoperative stay than thoracotomy, with equivalent oncological outcomes.

Disadvantages of robotic surgery are high capital costs, steep learning curve for the entire surgical team, inability to palpate, and (often) inability to obtain adequate robotic time in many hospitals.

Personal experience of 353 consecutive robotic anatomic lung resections was presented. A four-robotic arm technique with no access incision was used that differed from the technique employed by other groups; it had the advantage that the surgeon could assist himself, thereby reducing the variability associated with different assistants. Eighty-eight percent of patients had a malignancy, from whom a median of 17 lymph nodes was removed with median hospital stay of 2 days. Forty-one cases were converted to open surgery: 10 for bleeding (1 patient required transfusion) but no conversions for bleeding in the last 100 patients. Thirty-day postoperative mortality was 0.25% and 90-day mortality was 0.5%. Patients reported a median pain score of 2/10 at the 3-week follow-up (2).

As regards the costs of robotic surgery for lung lobectomy, an analysis of Medicare patients was made based on the State of Alabama’s Medicare reimbursement for lobectomy/segmentectomy of $18,937, which was considerably lower than for most other states of the United States. The median hospital charge was $32,000 per patient, direct costs were $13,800 per patient, so the institutional profit was $4,750 per patient. The calculation considered direct costs (items used in patient care) and indirect costs (overheads, including cost of the building and amortization of capital equipment and supplies, equipment maintenance, utilities and administrative staff costs). However, it was difficult if not impossible to factor in all contributors to costs (3).

## Robotic Lung Segmentectomy

While lobectomy remains the standard for stage I non-small cell lung cancer (NSCLC), low-dose CT screening is diagnosing more patients with small early-stage disease, who might be adequately treated by sublobar resection (4), and this is being investigated by ongoing trials (5). Segmentectomy has generally been performed by an open approach, and few publications on segmentectomy performed by VATS are available because it is a technically demanding procedure that until recently was performed by a small number of highly experienced groups (6).

However, since the advent of robotic surgery, several papers on robotic approaches to segmental dissection have been published (7–10). These indicate that robot-assisted thoracoscopic segmentectomy for malignant and benign lesions is practical, safe, and associated with few complications, short postoperative hospitalization, and adequate number of lymph nodes removed.

When performing a segmentectomy with a da Vinci robotic system, operating room staff set up the robot console, and the visual system. After induction of general anesthesia, the patient is placed in lateral decubitus with the hips flexed and secured. A double-lumen endotracheal tube is used to ventilate the lung not being operated on. The robot is positioned at the head or at the side of the patient. The operation begins with a 1-cm incision through the eighth intercostal space at the level of the mid-axillary line, through which the 30° high-definition stereoscopic camera is inserted to explore the thoracic cavity and provide visual guidance for the successive 3-cm utility incision, which is through the fourth or fifth intercostal space anteriorly. This is followed by an 8-mm incision at the eighth intercostal space in the posterior axillary line for the right robotic arm (on the right side) and another incision in the auscultatory triangle posterior for the final robotic arm. This fourth incision makes it possible to retract the lung and therefore better expose the operating field. The camera port is placed more laterally for the left side so that the heart does not obscure the operating field; otherwise, port placement does not vary. Dr. Cariboni went on to describe specific segmentectomies.

## Robotic Lung Lobectomy with Lymph Node Dissection

Robotic lobectomy plus lymph node dissection for lung cancer is performed in Milan using a four-arm system – three robot arm ports and a utility incision (mainly to remove the specimen) and no CO_2_ insufflation. To be eligible for robotic lobectomy patients had to have adequate cardiopulmonary reserve and a resectable lesion. The same extensive staging as for open surgery is performed (10, 11). The patient is positioned in lateral decubitus and single-lung anesthesia induced *via* a double-lumen endotracheal tube. The da Vinci Si robot is positioned slightly behind the patient’s head, while the da Vinci Xi robot is positioned lateral to the patient. Port positions are as described for segmentectomy and are standard for all lobectomies except that on the right side, the camera port through the seventh intercostal space is in the mid-axillary line, whereas on the left side, this port is moved 2 cm posteriorly so that the heart does not obscure vision of hilar structures. Lobectomy begins by isolating hilar elements from anterior to posterior using a hook and two Cadière graspers. One is used to retract the lung and expose structures; the other, manipulated by the left arm of the robot, and used to grip structures during dissection, is introduced through the utility incision for left side lobectomies or through the posterior eighth intercostal space port for right side lobectomies. Blood vessels and the bronchus are sectioned using mechanical staplers introduced by the assistant after removal of a robotic arm. The pulmonary vein is usually the first structure to be isolated and divided. If the lesion is in the right upper lobe, vein resection is followed by isolation of the branches of the pulmonary artery and sectioning, followed by isolation of the bronchus and bronchus sectioning. If the lesion is in the right lower lobe or left lung, after pulmonary vein sectioning, the bronchus is usually isolated and stapled before the artery. When performing middle lobectomy, the most favorable sequence is vein, bronchus, and artery.

Suspicious lymph nodes are usually removed before lobectomy, but radical lymph node dissection is performed after lobectomy. Paratracheal lymph node dissection is performed on the right side without azygos vein division. The mediastinal pleura between the superior vena cava and the azygos vein is incised. The lymph nodes, together with the fatty soft tissue of the right paratracheal space, are removed en bloc using a hook and Cadière grasper. In patients with large quantities of mediastinal fat or very large lymph nodes, an UltraCision harmonic scalpel is often used.

The nodes of the subcarinal station are removed after resection of the pulmonary ligament and retraction of the lung toward the anterior mediastinum, to expose the posterior mediastinum. Bronchial arteries are usually avoided without difficulty thanks to good visibility; otherwise, they can be coagulated. TachoSil sealant is sometimes applied to the fissure surface to reduce air leakage. A single 28 Ch pleural drain is positioned at the end of the operation.

While lymphadenectomy with VATS can be suboptimal and challenging (12), it is relatively easy to perform a radical dissection of the mediastinal and hilar lymph nodes using the robot (13).

## Robot-Assisted Thymectomy

Radical total thymectomy is the most effective means of achieving long-term remission in many patients with myasthenia gravis (MG) and is the standard treatment for localized thymoma. Since MG commonly affects young women, less invasive transcervical approaches were developed early, in opposition to transsternal approaches. The introduction of robotic-assisted thymectomy is rendering both these approaches obsolete. The first robotic excision of a thymoma was performed in 2001, since then, robotic thymectomy as treatment for thymoma and MG has expended rapidly. Over 100 centers worldwide perform robotic thymectomies, and approximately 3,500 robotic thymectomies were performed between 2001 and 2012 (14). MG remission rates and thymoma recurrence rates are comparable to those obtained with open procedures and perioperative complication rates are low (below 2%) (15). The group at the Charité University of Medicine in Berlin developed a widely used three-trocar unilateral robot-assisted thymectomy using the da Vinci robot system (14), in which all three trocars are placed along the submammary fold in women. Benefits for patients who undergo robotic thymectomy include the unilateral as opposed to bilateral approach of VATS (16) and the higher remission rates after robotic procedures than VATS (14).

## Minimally Invasive Surgery for Esophageal Cancer

Technical aspects of robotic esophagectomy were discussed, illustrated by video recordings showing details of port placement and the operation itself (17). Outcomes for 85 consecutive esophageal cancer patients were presented. These patients were scheduled for minimally invasive Ivor Lewis esophagectomy (laparoscopic or robotic abdominal and robotic chest). Sixty-four patients (75%) had preoperative chemoradiotherapy, 99% had esophageal cancer, and 99% had an R0 resection. There were no abdominal or thoracic conversions for bleeding. There was one abdominal conversion for inability to completely staple the gastric conduit. Mean operating time was about 6 h, median blood loss was 35 ml (no intraoperative transfusions), median number of resected lymph nodes was 22, and median length of stay was 8 days. Conduit complications (anastomotic leak or conduit ischemia) occurred in six patients. Thirty-day and 90-day mortalities were 3.5 and 10.6%, respectively. Poor initial results resulted in longer rehabilitation prior to surgery, liver biopsy in patients with suspected cirrhosis, and refinements to conduit preparation and anastomotic technique. Mortality was nevertheless disappointingly high, perhaps in part because of the high proportion who received induction chemoradiotherapy, although a previous report had 5.3% postoperative mortality in patients receiving induction therapy (18). It is also noteworthy that 30-day mortality was 0% and 90-day mortality was 7.1% for the last third of the series, so surgeon experience may have played a role.

## Minimally Invasive Esophagectomy for Cancer

Radical esophagectomy for cancer carries high risks of postoperative morbidity and mortality, since it requires violating both the abdominal and thoracic cavities. Recent case series, systematic reviews, and meta-analyses (19–21) clearly indicate that minimally invasive approaches to esophagectomy for cancer are associated with reduced in-hospital mortality, reduced incidence of pulmonary complications, lower intensive care requirements, shorter duration of hospital stay, reduced pain, and at least similar oncological outcomes to open surgery.

A randomized trial comparing quality of life and late complications in minimally invasive esophagectomy and open esophagectomy groups (22) showed, a year after surgery, that the physical health composite of the SF36 instrument, global health of the EORTC C30 questionnaire, and pain in the OES 18 questionnaire were significantly better in the minimally invasive group, while there were no differences in 1-year survival or complications. On the negative side, surgeons needed to be highly experienced in both esophageal surgery and minimally invasive techniques in order to obtain good results, and operating times were longer for minimally invasive than open surgery. Notwithstanding this, 30% of esophageal resections were recently reported a being performed using a minimally invasive approach (23). A 2015 paper from Palazzo et al. (24) compared a contemporary series of 104 patients who received minimally invasive esophagectomy, with 68 patients who received either open or hybrid esophagectomy from 2000 to 2013. The groups were of similar age and sex distribution. The minimally invasive esophagectomy group had lower operative mortality (3.9 versus 8.8%, *p* = 0.35) and significantly fewer major complications. Five-year survival was 64% in the minimally invasive group and 35% in the open group (*p* < 0.001). Multivariate analysis adjusted for covariates showed significantly worse survival in the open group.

These findings are in line with those of a 2015 paper from the UK (25), which used multivariate analysis to compare 83 patients given open esophagectomy, 187 patients given hybrid esophagectomy, and 64 patients given minimally invasive esophagectomy. Factors that correlated independently and significantly with survival were stage, nodal status, and type of operation (with minimally invasive patients surviving longer).

Taken together, these data suggest that totally minimally invasive esophagectomy is superior in several respects to open and hybrid techniques. However, even in many high volume centers, minimally invasive Ivor Lewis esophagectomy does not appear to be the technique of choice, perhaps because of difficulties with the anastomoses. For two-hole minimally invasive esophagectomy, several anastomotic techniques have been described including semi-mechanical anastomosis with a linear stapler and mechanical anastomosis with a circular stapler and a double-stapling technique. No technique appears superior, but good results have also been reported for anastomoses performed robotically, suggesting that availability of a robot might encourage more widespread use of minimally invasive esophagectomy.

## International Network for Robotic Thoracic Surgery

Proposals for an international network of robotic thoracic surgeons were presented. The network, to be called Robotic Chest Surgeons Network (ROCS-NET) aims to promote the safe use of robotic equipment in thoracic surgery, in a context in which many surgeons still prefer open posterolateral or muscle-sparing thoracotomies, because of the disadvantages to the surgeon of performing VATS. Robotic surgery has several advantages (intuitive movements, tremor filtration, more degrees of manipulative freedom, motion scaling, high-definition stereoscopic vision, stable camera platform, equivalence between dominant and non-dominant hands, and eye–hand–target alignment) (11–17) that should make it more accessible than VATS, both to trainees and experienced thoracic surgeons, and also expand indications. Furthermore, new and improved robotic devices are likely to become available soon, encouraging the expansion of robotic surgery and hopefully reducing costs.

The Robotic Chest Surgeons Network will be concerned with surgeon training and quality control so as to minimize complications during learning, including in particular: (a) standardization and certification of training; (b) monitoring adverse events and developing quality performance measures; (c) holding master classes to train young thoracic surgeons in robotic skills; (d) data sharing (common database) and facilitating clinical research; and (e) fostering a climate of cooperation to encourage researchers to exchange ideas and design trials.

The Network will also contribute to the development of thoracic health care policies.

Few organizations concerned specifically with robotic surgery exist. One of the most important is the Clinical Robotic Surgical Association (CRSA) founded in Chicago in 2008. However, this society does not attract enough European thoracic surgeons because it mainly focuses on abdominal diseases and because most of its conferences are held in the US. ROCS-NET is specifically aimed at thoracic surgeons, at all levels, specializing in robotic surgery.

ROCS-NET will incorporate and develop an autonomous nodes network (ANN) – an information system in which every node in the network is autonomous (responsible for its own data), overcoming the fear that many investigators have of loosing control of their data when they enter multicenter studies, although it can share data with the other nodes. ANN in fact would be a decentralized IT system that encourages spontaneous studies from individual participating centers.

## The Costs of Robotic Surgery

Experience of robotic surgery at the Pisa Multidisciplinary Robotic Center was analyzed. The Center was set up in 2009 and by 2015 had 36 surgeons performing 38 types of surgical procedure, using three da Vinci robots. A thousand robotic surgical procedures were performed in 2015. Management’s aim was to achieve sustainability based on high surgical volumes, the performance of complex procedures, and standardization. To identify ways of achieving greater efficiency, intensive monitoring of activity was undertaken which resulted in major modifications to working practice. A Bill of Materials (BoM) was generated for every surgical procedure performed by each surgeon so as to identify all items of cost and also variations in behavior. Implementation of protocols helped to effectively contrast unnecessary variations in practice and achieve standardization.

After 6 years of experience, positive effects of standardization and monitoring using the BoM system had become evident, and for some robotic surgical procedures (including lung lobectomy) analyses that considered revenues from disease-related groups (DRGs) and total activities-based costs, indicated that the Center was making a profit.

It was found that savings were not obtained directly from innovation but by using innovation as a starting point to stimulate more efficient organization. As a university and academic hospital, there was a natural propensity to innovate, which helped the hospital to better meet the needs of the public. The Center was also using surgical robotic as an integral part of its surgeon training programs.

## Robotic Surgery Versus Vats for Major Lung Resections: A Prospective Randomized Trial

A randomized trial (NCT02804893) to compare VATS with robotic surgery for lung resections to treat lung cancer is about to start recruiting. VATS is an established approach to resectable lung cancer, being associated with reduced pain, fewer complications, shorter postoperative stay, and equivalent oncological outcomes compared to thoracotomy (1, 26). Furthermore, its use is expanding rapidly: in 2015, 50% of the pulmonary lobectomies performed in the United States, and 33% of those performed in Europe employed a minimally invasive approach (27) However, because of restricted freedom of movement and poor ergonomics, VATS is demanding for thoracic surgeons and requires a long learning curve.

In theory, robotic surgery overcomes many of the disadvantages of VATS, but these benefits have not been conclusively demonstrated. A recent large retrospective comparison of the two techniques (28) found that for robotic lobectomy and wedge resection, costs were higher and operating times longer than for VATS, with no difference in adverse events. However, 40 robotic centers were included in the evaluation – an average of 8 robotic cases per center, suggesting that many of the surgeons may still have been learning the robotic technique.

A smaller 2015 comparison (29) of the first 28 VATS lobectomies with the first 28 robotic lobectomies performed in a single institution reported that perioperative outcomes were similar for both techniques during the learning period but that the robotic approach was associated with better safety and fewer conversions for uncontrolled bleeding.

NCT02804893 is a prospective multicentric randomized trial to compare complications and conversion rates between robotic and VATS approaches to stage I and II lung cancer. It is being coordinated by Humanitas Research Hospital, Rozzano, Italy, and so far, institutes from Istanbul, Marseilles, Strasburg, and Florida have indicated their willingness to join. The primary endpoint is a combination of adverse events (intraoperative and postoperative complications, conversions). Secondary endpoints are duration of surgery, number of resected lymph nodes, hospital stay, pain, quality of life, immune response, and respiratory function. Inclusion criteria are age >18 years, known or suspected lung cancer, clinical stage T1–T2, N0–N1, patient candidate for lobectomy or anatomical segmentectomy, and good general condition (American Society of Anesthesiologists Physical Status I or II). Exclusion criteria are metastatic cancer, extrapulmonary primary cancer, severe heart disease, alcohol abuse, renal impairment, or presence of other serious comorbidities.

Any intraoperative/perioperative complication or conversion will be regarded as a failure of surgery. The trial will need to recruit 150 patients per arm to have an 80% power (5% significance level) to demonstrate a reduction of 15% in surgery failures, assuming 35% failures with VATS and 20% with robotic surgery. This sample size also has 80% power to detect a 15% reduction in operating time (from 210 to 180 min), 95% power to detect a difference in blood loss, and 95% power to detect a difference in mean number of mediastinal lymph node stations dissected.

## New Developments in Computer-Assisted Surgery

Promising new developments in computer-assisted surgery rely on modern imaging technology in combination with virtual reality and augmented reality. Three main steps are involved: (a) obtaining a 3D model of the patient’s anatomy from his/her CT or MRI scans; (b) use of this patient-specific model (virtual reality model) for preoperative surgical planning and surgical simulations; and (c) superimposition of this model onto the intraoperative view of the patient provided by the robotic visual system. This third step is augmented reality: it is still undergoing intensive development but is currently capable of providing the surgeon with a superimposed transparent view of the patient’s internal anatomy, resulting in improved lesion targeting, improved ability to identify critical structures such as blood vessels that are not visible in the surgeon’s real field of view, and better tracking of instruments. Initially augmented reality relied on a non-modifiable virtual reality model of the patient – one that could not take organ deformation into account. However, the latest generation of augmented reality overcomes this limitation by using intraoperative 3D image acquisition and a non-rigid temporal registration algorithm (30).

The near future development of the technology is expected to involve “robotizing” the procedures or replacing many the surgeon’s maneuvers by automated or robotic maneuvers. This implies extensive further development, so that it will be able to give the real-time position and shape of organs, blood vessels, and diseased structures.

## Medical Applications of Robots

The humanoid robot called Icube was developed at the Robotics Department of the Italian Institute of Technology (IIT). The development of Icube and its sub-systems has driven, and continues to drive, advances in medical prosthetics and medical robotics. The robot is comparable in height to a 6-year-old child, which however would run all day on a bar of chocolate, while Icube would only last 10 min on that amount of energy. Furthermore, Icube only has a 2D brain; nonetheless, it is impressive. Its skin has over 4,000 contact points and sensors, so it can sense contact and contact forces and hence “understand” if it is being pulled or pushed and respond accordingly to maintain equilibrium. This haptic feedback system allows Icube to adapt and interact, allowing it to be a social humanoid. In fact, it has a vestibular system incorporating gyroscopes and sensors. The sensors are copied from nature. In nature, hair cells transform sounds (or mechanical vibrations in fish) into electric fields. The robot has the equivalent: Icube’s skin is made of a high technology plastic that is sensitive to tactile stimuli. For example, the skin is sensitive to being blown on, but it also transforms that “wind” energy – 15 μW – into an electric current whose energy can be used. This secret of the plastic skin is that it incorporates conductive circuits made of graphene, which are printed onto the plastic using a 3D printer and graphene ink. Graphene is a Nobel-prize-winning 2D material that is cheap, ultra-light, 200 times stronger than steel, flexible, thin, transparent, and a superb conductor. Graphene ink is incorporated into sophisticated prostheses for amputees.

One example of such a prosthesis is a completely 3D-printed prosthetic hand with a single 9-W motor and a triple tendon system able to make 85% of the “grasps” of a human hand. It receives signals from muscle movement sensors on the patient’s arm and has an artificial intelligence system to translate signals into prosthetic hand movements (31). After only 2 months of rehabilitation and training, a patient can use the hand to perform manual work and execute fine movements. The hand currently costs 10,000 euro and is being tested on hundreds of patients.

Microtechnology and Systems for Robot-Assisted Laser Phonomicrosurgery is a European project coordinated by the IIT, which is enabling surgeons to better manipulate the cutting instrument (laser scalpel) when performing vocal cord surgery. The fully integrated robot has a laser head and periscope controlled by high precision piezoelectric stage, with illumination. The surgeon wears an oculus to visualize the operating field from a laryngeal surgery endoscope. Magnification can be changed at will. Virtual reality is also projected by the oculus onto the surgeon’s field of view. Operating in virtual reality connected to a graph-pad with a pen, the surgeon uses the pen of the graph-pad to do what would normally be done with the scalpel. The real laser scalpel can then be instructed to automatically make the cut planned by the surgeon on the graph-pad, with very high precision laterally (4 μm) and in depth (120 μm). Protected areas (blood vessels, nerves) can be defined. The latest robot for laryngeal microsurgery is called μRALP or micro-RALP (http://www.microralp.eu/).

The micro-robot called Plantoid is a novel robotic system that can “grow” into the body to reach a predetermined objective (like a root grows out from a plant into the soil) carrying with it sensors, biomarkers, or therapeutic agents. Plantoid grows in the “right” direction (avoiding critical structures) because it has sensors that allow it to avoid obstacles, hard surfaces, and high temperatures, etc. The tip is able to grow into the body to the desired target while remaining connected to its source outside the body, because the materials for growth are provided continuously as in 3D printing.

## Conclusion

Minimally invasive approaches to thoracic oncologic surgery are fast becoming mainstream. Large proportions of lobectomies in the United States and Europe (27), and increasing proportions of esophagectomies and thymectomies, are now being performed using these techniques. This contrasts with the situation in 2008 when a survey of European surgeons found that nearly 60% of responders used VATS for lobectomy in less than 5% of cases (32) Thus, it would appear that the established advantages of minimally invasive over open surgery – particularly shorter hospital stay, reduced long- and short-term morbidity, and at least equivalent oncological outcomes – are prompting the switch to VATS, notwithstanding its inherent difficulties for the surgeon.

Robotic thoracic surgery is also gaining in popularity. Surgeons who presented their experience at this Workshop were enthusiastic about robots and unanimous in their opinion that the robotic approach to the thorax was easier for the surgeon than VATS. The Workshop also highlighted current problems with robotic surgery, in particular high capital and running costs, and lack of high quality evidence that robotic surgery is superior to VATS in terms complications, conversion rates, and oncological radicality [although recent data suggest that robotic surgery is superior to VATS for mediastinal lymph node dissection (13)]. It is encouraging therefore that a multicentric randomized trial comparing robotic and VATS approaches to stages I and II lung cancer is about to start. Robotic surgery is a fast-developing field, and numerous innovations and improvements can be expected in the near future. Perhaps the most exciting of these is the use of modern imaging technology to produce augmented reality representations of the patient’s anatomy, which can be superimposed onto the intraoperative view of the patient provided by the robotic visual system, to improve lesion targeting and identification of critical structures.

## Author Contributions

MA, as chief of the thoracic surgery of Humanitas Hospital, coordinated the conference and reviewed and approved the paper. UC was responsible for the description of the indications for and results of robotic segmentectomy for lung cancer. EB described the technique and reviewed the literature on manual videothoracoscopic thymectomy for thymoma and myasthenia. RC described the reasons for and difficulties in starting a robotic program in thoracic surgery, and described the technique and results of his experience in robotic lobectomy and robotic esophagectomy. RC, as Scientific Director of the IIT, described the development of three projects related to robotic systems. UF described the technique of videothoracoscopic esophagectomy and reviewed the literature. CM reported on a multidisciplinary robotic program at Pisa from the perspective of an administrator. JR described technique and results for a robotic approach to mediastinal diseases at his center. LS described the integration of robotic surgery with imaging technology focusing on the potential of virtual reality applied to surgical disciplines. AT was involved in discussions on future research programs in robotic thoracic surgery. RC responsible for the Sections ROBOTIC SURGERY FOR LUNG RESECTIONS and MINIMALLY INVASIVE SURGERY FOR ESOPHAGEAL CANCER; UC and GV responsible for the Section ROBOTIC LUNG SEGMENTECTOMY; GV responsible for the Sections ROBOTIC LUNG LOBECTOMY WITH LYMPH NODE DISSECTION and ROBOTIC SURGERY VERSUS VATS FOR MAJOR LUNG RESECTIONS: A PROSPECTIVE RANDOMIZED TRIAL; JR responsible for the Section ROBOT-ASSISTED THYMECTOMY; UF responsible for the Section MINIMALLY INVASIVE ESOPHAGECTOMY FOR CANCER; GV and AT responsible for the Section INTERNATIONAL NETWORK FOR ROBOTIC THORACIC SURGERY; CM responsible for the Section THE COSTS OF ROBOTIC SURGERY; LS responsible for the Section NEW DEVELOPMENTS IN COMPUTER-ASSISTED SURGERY; RC responsible for the Section MEDICAL APPLICATIONS OF ROBOTS.

## Conflict of Interest Statement

Giulia Veronesi has received an honorarium for one activity as proctor of robotic surgery from ABI Medica. The remaining authors declare no conflict of interest.
